# Pentraxin-3 is not related to disease severity in cirrhosis and hepatocellular carcinoma patients

**DOI:** 10.1007/s10238-020-00617-4

**Published:** 2020-02-20

**Authors:** Susanne Feder, Elisabeth M. Haberl, Marlen Spirk, Thomas S. Weiss, Reiner Wiest, Christa Buechler

**Affiliations:** 1 Department of Internal Medicine I, Regensburg University Hospital, 93042 Regensburg, Germany; 2 Children’s University Hospital (KUNO), Regensburg University Hospital, Regensburg, Germany; 3 Department of Visceral Surgery and Medicine, University Inselspital, Bern, Switzerland

**Keywords:** MELD score, Ascites, Prothrombin time, Varices, Liver cancer

## Abstract

The acute-phase protein pentraxin-3 (PTX3) is a component of the innate immune system. Inflammation and tissue injury increased PTX3 in the injured liver, and accordingly, circulating PTX3 was induced in patients with chronic liver diseases. In the present study, PTX3 protein was determined in systemic, hepatic, and portal vein plasma of patients with liver cirrhosis to assess a possible association between hepatic PTX3 release and extent of liver injury. However, PTX3 levels were not related to disease severity. Of note, portal PTX3 levels were higher than concentrations in the hepatic vein. PTX3 in the hepatic and portal veins was negatively correlated with factor V, antithrombin 3, and prothrombin time. PTX3 did neither correlate with C-reactive protein nor galectin-3 or resistin, whereby the latter two proteins are associated with hepatic injury. PTX3 levels were not changed in cirrhosis patients with ascites or varices and did not correlate with the hepatic venous pressure gradient. Likewise, serum PTX3 was not correlated with histological steatosis, inflammation, or fibrosis stage in patients with hepatocellular carcinoma (HCC). Moreover, PTX3 was not associated with tumor node metastasis classification in HCC. Above all, PTX3 increased in hepatic, portal, and systemic blood immediately after transjugular intrahepatic portosystemic shunt (TIPS). Higher PTX3 in portal than hepatic vein plasma and further increase after TIPS suggests that the liver eliminates PTX3 from the circulation. In summary, PTX3 is not of diagnostic value in cirrhosis and HCC patients.

## Introduction

Liver cirrhosis is the advanced stage of all chronic liver diseases [1]. Common complications are ascites, esophageal varices, and renal impairment [2]. Portal hypertension accounts for these severe consequences [3]. Transjugular intrahepatic portosystemic shunt (TIPS) lowers portal pressure and is a valuable option for the treatment of patients with refractory ascites and bleeding esophageal varices [4]. Adverse events of TIPS are hepatic encephalopathy [4] and thrombosis [5]. Liver tumors are considered a contraindication for TIPS, and this intervention was discussed to promote the onset of hepatocellular carcinoma (HCC) [6].

Pentraxin 3 (PTX3) is an acute-phase protein, and thus its expression is strongly induced in inflamed and injured tissues. Unlike C-reactive protein (CRP), which is primarily synthesized in hepatocytes, PTX3 secretion was increased in hepatic stellate cells, neutrophils, and monocytes [7]. Impaired hepatocyte function and accordingly CRP synthesis limit the diagnostic value of CRP in patients with decompensated liver cirrhosis [8]. PTX3 is a reliable marker for the activation of immune cells and was supposed to be superior to CRP in these patients [9].

PTX3 has an important function in tissue repair and wound healing. PTX3 deficiency was associated with increased clotting and fibrin deposition in line with a role of PTX3 in plasmin-mediated fibrinolysis [10, 11]. Hepatic stellate cells are the main cells responsible for tissue repair in the liver [12]. Accordingly, in experimental models of liver injury, PTX3 expression increased in these cells and contributed to enhanced synthesis of extracellular matrix proteins like collagens. Moreover, hepatic inflammation and injury improved [11].

The prevalence of the metabolic syndrome, which is a risk factor for non-alcoholic fatty liver disease (NAFLD), is increasing. Plasma PTX3 was induced in patients with the metabolic syndrome. Of note, PTX3 was negatively correlated with high density lipoprotein [13]. A further study described a positive association of PTX3 mRNA expression in white blood cells with low-density lipoprotein [13, 14]. Accordingly, serum PTX3 was positively correlated with LDL-cholesterol in a cohort of type 2 diabetes patients [15]. Notably, type 2 diabetes patients with NAFLD had serum PTX3 levels as high as patients with normal liver function illustrating an association of serum PTX3 with dyslipidemia rather than NAFLD [13, 15]. Anyhow, a separate study described an association of plasma PTX3 with the stages of liver fibrosis in patients with NAFLD [16]. Moreover, PTX3 was strongly induced in alcoholic liver disease and was related to the model for end-stage liver disease (MELD) score. Plasma PTX3 was positively correlated with hepatic PTX3 gene expression indicating that the liver contributed to higher systemic levels [11]. In hepatitis C virus (HCV)-infected patients, plasma PTX3 did not differentiate mild from severe fibrosis [17]. Accuracy of fibrosis assessment in HCV-infected patients was nevertheless improved by a score combining PTX3 levels, gamma-glutamyl transpeptidase/platelet count ratio, hyaluronic acid, and age [18].

The prevailing opinion nowadays is that systemic PTX3 is induced in patients with chronic liver diseases [11, 19] and has a positive predictive value for adverse clinical outcomes [20, 21]. Liver cirrhosis is a risk factor for HCC, and PTX3 may also have a role herein [17, 22].

Inflammation is a critical component of carcinogenesis and drugs which boost the anti-tumor immune response were successful in cancer therapy [23]. Genetic diversity of killer-cell immunoglobulin-like receptor and human leukocyte antigen was related to the risk of HCV-related HCC further highlighting the role of the innate immune system in cancer [24]. Indeed, PTX3 facilitated progression of different cancers, and PTX3 expression in hepatocellular carcinoma (HCC) tissues was positively associated with shorter survival time of the patients [19]. Accordingly, plasma PTX3 was high in HCC compared to patients with severe liver fibrosis [17]. Cancer-associated fibroblasts support tumor growth and metastatic disease. These cells were characterized by a high expression of profibrotic and proinflammatory genes and suppression of PTX3 [25]. PTX3 deficiency was linked to cancer-related inflammation, angiogenesis, and mutations in models of skin cancer [22]. Whether PTX3 functions as a tumor-suppressor or tumor-promoting factor in HCC requires future work.

In this study, we hypothesized that induced PTX3 synthesis in the cirrhotic liver results in higher PTX3 protein levels in the hepatic vein. Moreover, we postulated positive correlations of hepatic vein PTX3 with measures of liver function in patients with liver cirrhosis and patients with HCC.

## Materials and methods

### Transjugular intrahepatic portosystemic shunt (TIPS)

The study included 35 patients with liver cirrhosis. Table 1 lists the laboratory values of the patients. The etiology of liver cirrhosis was alcoholic in 30 patients, hepatitis C infection in 2 patients, and of other reasons in 3 patients. Transjugular intrahepatic portosystemic shunt (TIPS) (Viatorr-Stent, Putzbrunn, Germany) implantation was done in the fasted state [26]. Complications requiring the insertion of the TIPS were variceal bleeding in 10 patients, hepatorenal syndrome in 1 patient, and refractory ascites in 24 patients. During this intervention, EDTA plasma of the hepatic vein (HVP), which was not drained by the TIPS stent, of the portal vein (PVP), and of a peripheral vein (SVP) was collected. Blood samples were available from 35 (HVP), 34 (PVP) and 26 (SVP) patients. Plasma was also obtained immediately after TIPS implantation, and HVP of 34, PVP of 35, and SVP of 34 patients were available for this study. Blood samples of these patients were used in previous studies [27, 28]. Routine laboratory parameters such as alanine aminotransferase and aspartate aminotransferase were measured by the Institute for Clinical Chemistry and Laboratory Medicine (University Hospital of Regensburg). The study was in accordance with the Declaration of Helsinki and was approved by the Ethical Committee of the University Hospital of Regensburg. All patients provided written informed consent.

**Table 1:** Patient demographics and laboratory parameters

	TIPS patients	HCC patients	*p* value
Number	35	31	
Sex (female/male)	9/26	5/26	
Age (years)	52 (40–81)	64 (33–85)	0.007
Child–Pugh stage A/B/C	9/12/14		
MELD score	8 (6–21)		
Ascites: no/little/modest/massive	4/9/3/19		
Variceal size: no/small/large	6/7/22		
C-reactive protein (mg/l)	13.4 (1.0–53.5)^32^		
Fibrinogen (mg/dl)	310 (114–520)^34^		
Antithrombin 3 (%)	64.4 (23.6–95.8)^31^		
Factor V (%)	59 (17–137)^25^		
ALT (U/l)	37 (4.0–108.0)	51 (17–378)^29^	
AST (U/l)	29.0 (2.0–82.0)	37 (14–502)^30^	
Albumin (g/l)	31.3 (1.6–47.0)		
Bilirubin (mg/dl)	1.1 (0.3–8.2)	0.6 (0.2–2.5)^30^	< 0.001
Quick prothrombin time (%)	72 (28–100)	92 (76–100)^23^	< 0.001
Creatinine (mg/dl)	1.0 (0.5–4.5)		

Median values and range of values were listed. Uppercase numbers were included when data were not available for the whole study group

*CRP* C-reactive protein, *ALT* alanine aminotransferase, *AST* aspartate aminotransferase

### Hepatocellular carcinoma

Serum of 31 HCC patients was also available to measure PTX3. Details of the cohort are listed in Table 1 and were described in detail recently [29]. Experiments adhered to the guidelines of the charitable state controlled Human Tissue and Cell Research foundation. Ethical approval was obtained from the ethical committee of the Regensburg University Hospital. Each patient signed a written informed consent form.

### Pentraxin-3 ELISA

The human pentraxin-3 DuoSet ELISA was from R&D Systems and was performed as recommended by the company (Wiesbaden, Nordenstadt, Germany). Plasma was diluted 1:2.5-fold for analysis.

This ELISA was used in a previous study where serum was analyzed. The intra-assay coefficient of variation (CV) for the PTX3 ELISA was 5%, and the total CV for the assay was 7%. There was, however, no detailed information about the methods applied to evaluate this ELISA [30]. Mean serum PTX3 levels were about 2 ng/ml in that study cohort [30]. In the current analysis, median total % CV was 7.3%. For samples with PTX3 levels below 2 ng/ml, the % CV was 3.2, and for samples with PTX levels above 5 ng/ml, the % CV was 6.7%. Mean inter-assay % CV (5 samples in 4 replicates) was 9.4%. These % CV values were within the recommended % CVs which are up to 10% for intra-assay and up to 15% for intra-assay variations [31].

Fetal calf serum (two different batches) was analyzed as negative control (4 replicates on 4 different days). Values were always below the detection limit of the ELISA.

Recovery was determined by the use of recombinant PTX3 (provided by the company to be used in the standard curve). Here, 5.6, 2.2, and 0.9 ng protein were spiked into plasma of at least three different donors. The % recovery was 81, 79, and 88, respectively. This is almost within the acceptance range of 80–120% [32].

### Statistics

Data are presented as box blots (IBM SPSS Statistics 25.0). Outliners were given as circles, (greater than 1.5 times the interquartile range) or stars (greater than 3.0 times the interquartile range). Statistical differences were analyzed by two-tailed Mann–Whitney *U* test. A *p* value < 0.05 was regarded as significant. Spearman correlation was calculated using the IBM SPSS Statistics 25.0 software.

## Results

### Pentraxin-3 in serum of patients with liver cirrhosis

**Fig. 1:**
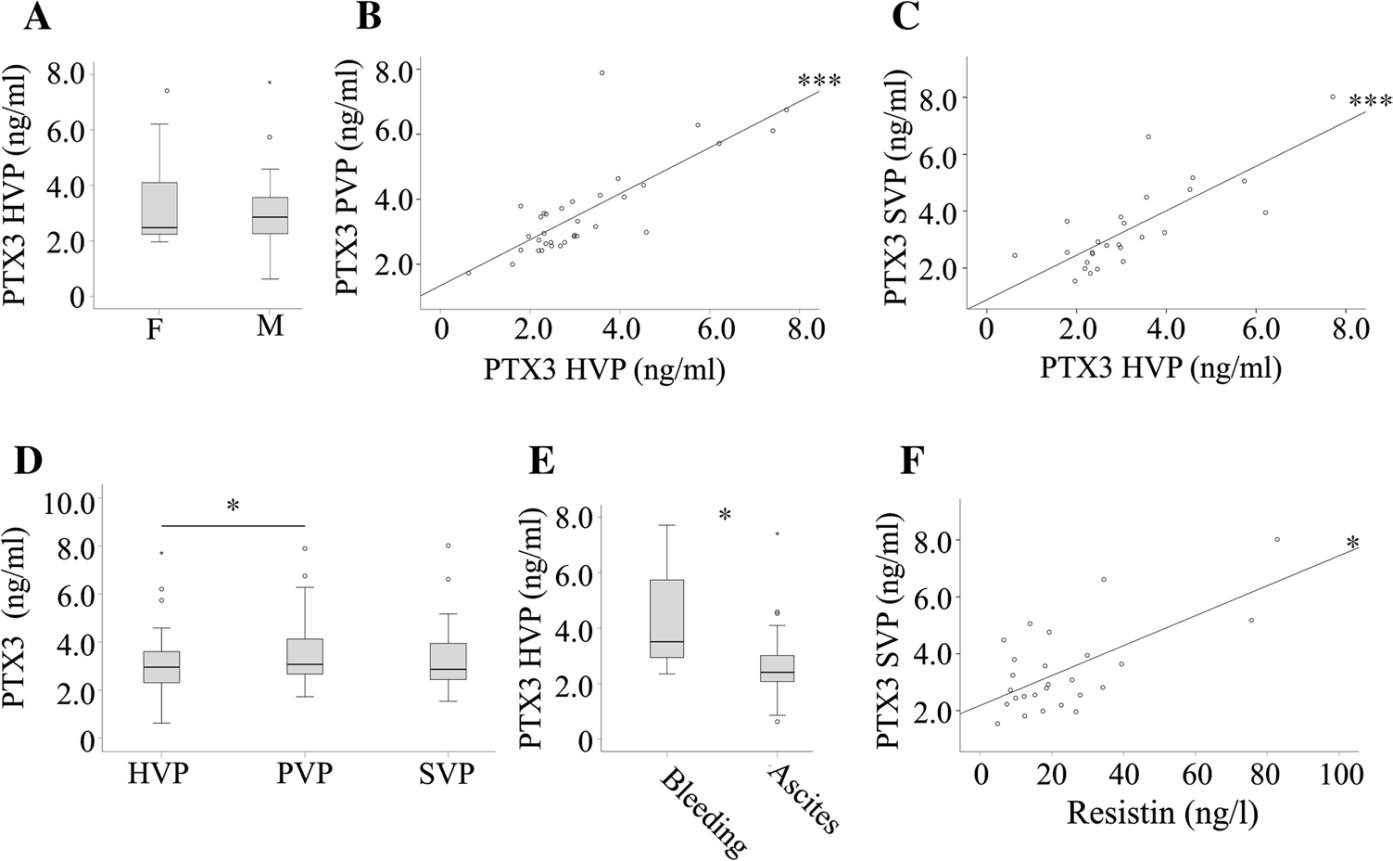
PTX3 in plasma of patients with liver cirrhosis. **a** PTX3 levels in females (F) and males (M). **b** Correlation of portal vein plasma (PVP) and hepatic vein plasma (HVP) PTX3. **c** Correlation of HVP and systemic vein plasma (SVP) PTX3. **d** PTX3 in HVP, PVP, and SVP. **e** HVP PTX3 in patients undergoing TIPS because of variceal bleeding or ascites. **f** Correlation of SVP PTX3 with resistin. *p < 0.05, ***p < 0.001

Thirty-five patients suffering from clinically diagnosed liver cirrhosis were enrolled in the study. Pentraxin-3 (PTX3) plasma concentrations were similar in both sexes (Fig. 1a). PTX3 did not correlate with the age of the patients (data not shown). Correlations were identified for systemic vein plasma (SVP) and portal vein plasma (PVP, *r* = 0.682, *p* < 0.001), SVP and hepatic vein plasma (HVP, *r* = 0.777, *p* < 0.001) and PVP and HVP PTX3 (*r* = 0.734, *p* < 0.001) (Fig. 1b, c and data not shown).

PTX3 was higher in PVP compared to HVP levels, which were similar to SVP concentrations (Fig. 1d). Indications for TIPS placement were mostly ascites and variceal bleeding. Latter group had higher PTX3 in HVP and a trend to increased levels in PVP (*p* = 0.051) (Fig. 1e and data not shown).

### Association with systemic inflammatory markers

PTX3 did not correlate with C-reactive protein (CRP) in the cirrhotic patients (Table 2). Moreover, there were no associations of PTX3 with proteins released by immune cells which were measured in the serum of these patients before [28, 33]. Galectin-3 acts as a regulatory molecule in acute and chronic inflammation [34], but did not correlate with PTX3 (Table 2). Resistin is mainly synthesized by macrophages [35] and was not associated with PTX3 in HVP and PVP. Positive correlations of PTX3 and resistin were found in SVP (Fig. 1f, Table 2).

**Table 2:** Correlation coefficients of PTX3 in the different blood compartments of cirrhosis patients with CRP measured in systemic blood, serum resistin, and serum galectin-3 measured in the respective blood compartments

PTX3	HVP	PVP	SVP
CRP (peripheral)	0.206 (32)	0.077 (31)	0.274 (25)
Resistin	0.236 (31)	0.208 (31)	0.418* (26)
Galectin-3	0.141 (31)	0.155 (31)	0.058 (26)

Numbers in brackets indicate the number of patients where these data were available. *p < 0.05

### Association of pentraxin-3 with the severity of liver cirrhosis

**Fig. 2:**
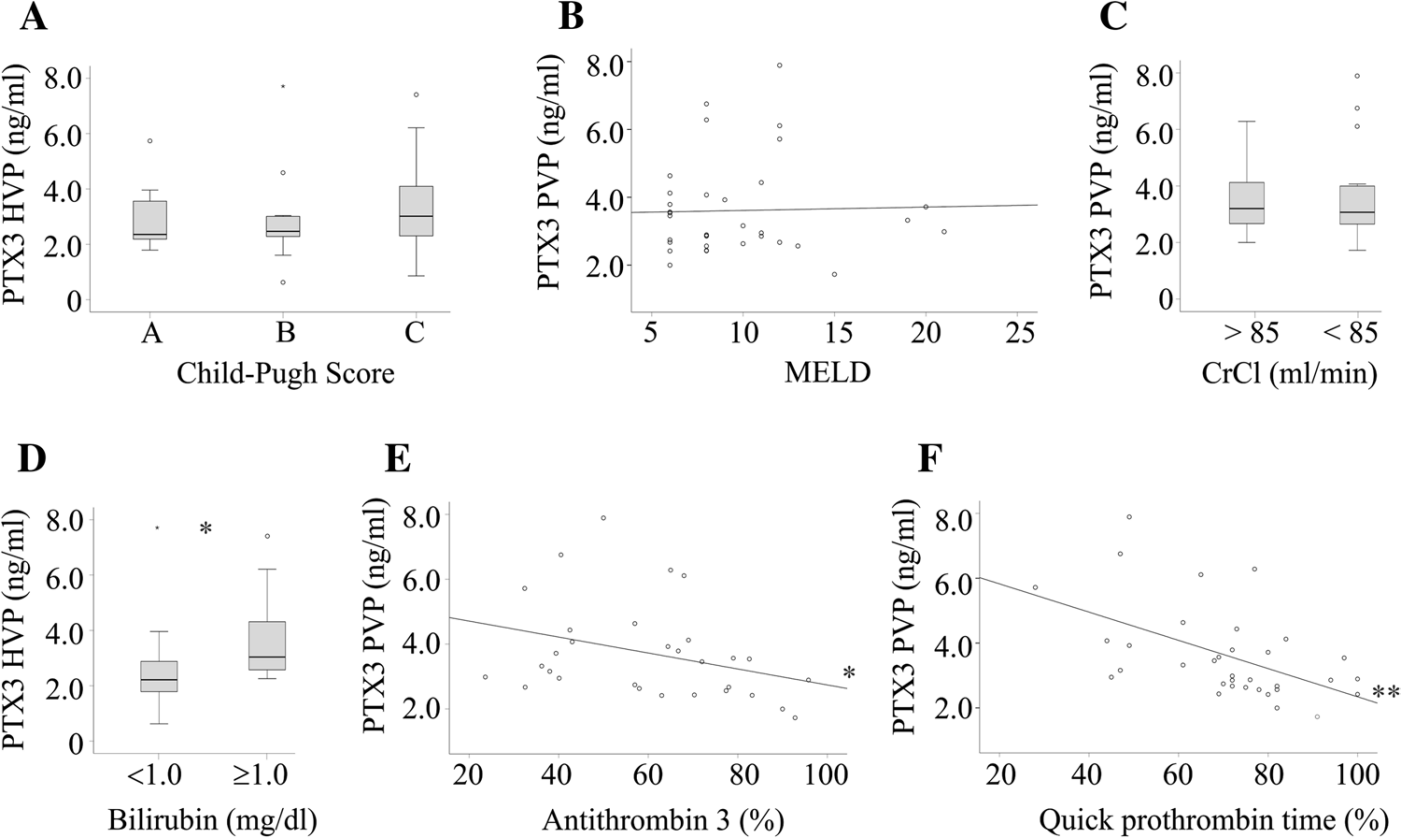
Associations of PTX3 with Child–Pugh and MELD score, creatinine clearance (CrCl), bilirubin, antithrombin 3, and prothrombin time. **a** PTX3 in hepatic vein plasma (HVP) of patients stratified for Child–Pugh score. **b** Correlation of portal vein plasma (PVP) PTX3 with the MELD score. **c** PVP PTX3 in patients with normal and impaired (< 85 ml/min) CrCl. **d** HVP PTX3 in patients with normal and high (≥ 1) bilirubin. **e** Correlation of PVP PTX3 with antithrombin 3. **f** Correlation of PVP PTX3 with quick prothrombin time. *p < 0.05, **p < 0.01

PTX3 was similar in patients with compensated and decompensated liver cirrhosis (Fig. 2a). Accordingly, levels in HVP, PVP, and SVP were not correlated with the MELD score (Fig. 2b and data not shown).

### Associations with markers of hepatic and kidney function

There were no correlations of HVP, PVP, and SVP PTX3 levels with albumin, aspartate aminotransferase, alanine aminotransferase, serum urea, creatinine, or creatinine clearance (data not shown). Accordingly, PTX3 levels were similar in patients with normal (85–160 ml/min, 14 patients) and impaired (20 patients) creatinine clearance (Fig. 2c). Bilirubin was positively associated with HVP PTX3, and thus, PTX3 was higher in patients with bilirubin levels ≥ 1 mg/dl (Fig. 2d, Table 3).

**Table 3:** Correlation coefficients of PTX3 in the different blood compartments of cirrhosis patients with bilirubin, prothrombin time, antithrombin 3, fibrinogen, and factor V analyzed in peripheral blood

PTX3	HVP	PVP	SVP
Bilirubin	0.447** (35)	0.196 (34)	0.141 (26)
Prothrombin time	− 0.467** (35)	− 0.528** (34)	− 0.292 (26)
Antithrombin 3	− 0.543** (31)	− 0.374* (30)	− 0.226 (22)
Fibrinogen	− 0.545** (34)	− 0.328 (33)	− 0.231 (25)
Factor V	− 0.403* (25)	− 0.489* (25)	− 0.327 (20)

Numbers in brackets indicate the number of patients where these data were available. *p < 0.05, **p < 0.01

Fibrinogen negatively correlated with HVP pentraxin-3 (Table 3). Antithrombin 3 and factor V were negatively associated with HVP and PVP PTX3 (Fig. 2e, Table 3). Further, quick prothrombin time was negatively correlated with HVP and PVP PTX3 (Fig. 2f, Table 3). PVP and HVP PTX3 were lower in patients with normal prothrombin time (22/21 patients, respectively) compared to those 13 patients with values ≤ 70% (prothrombin time > 70% is the normal range) (data not shown).

### Ascites and varices

**Fig. 3:**
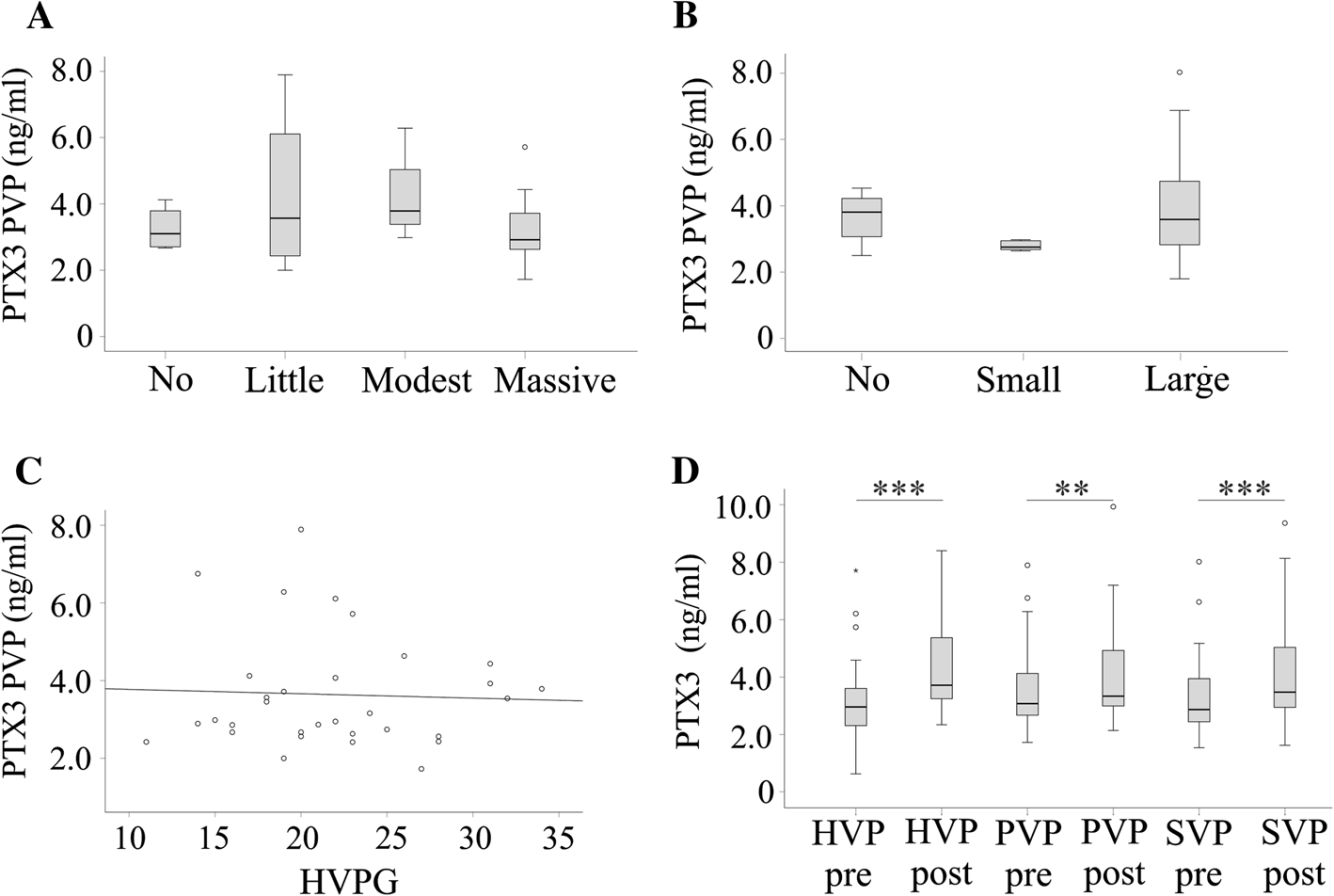
Associations of PTX3 with ascites, varices, and hepatic venous pressure gradient (HVPG, which is the gradient between pressures in the portal vein and the inferior vena cava) and levels of PTX3 shortly after TIPS. **a** Portal vein plasma (PVP) PTX3 in patients without, little, modest and massive ascites. **b** PVP PTX3 in patients without, with small and large varices. **c** Correlation of portal vein plasma (PVP) PTX3 ratio with HVPG. **d** PTX3 in the three compartments before and shortly after TIPS. Significant higher level of PVP compared to HVP PTX3 before TIPS was not marked in this figure. **p < 0.01, ***p < 0.001

PTX3 was neither associated with ascites nor with variceal size (Fig. 3a, b). The hepatic venous pressure gradient (HVPG) is linked to the formation of ascites and varices [36] and was known from 33 patients. PTX3 in any blood compartment did not correlate with HVPG (Fig. 3c and data not shown).

### PTX3 shortly after TIPS

PTX3 was also measured in the respective blood compartments immediately after TIPS. PTX3 levels were significantly higher after stent insertion in portal, hepatic, and systemic venous plasma. Post-TIPS PTX3 was similarly concentrated in all of these compartments (Fig. 3d). Increase in HVP PTX3 was 132.3 (86.8–220.0) % and was significantly higher than rise in PVP level, which was 107.3 (80.0–179.3) % (*p* = 0.012). SVP increase was 122.5 (72.9–182.8) % and was similar to changes in the two other blood compartments.

After TIPS, antithrombin 3 was negatively associated with PTX3 in PVP (Table 4). Prothrombin time and factor V were negatively correlated with HVP and PVP PTX3 levels (Table 4). Associations of post-TIPS PTX3 with fibrinogen and bilirubin did not exist (Table 4). PTX3 in any compartment after TIPS did not correlate with pre-TIPS CRP, galectin-3, or resistin levels (data not shown).

**Table 4:** Correlation coefficients of post-TIPs PTX3 in the different blood compartments with bilirubin, prothrombin time, antithrombin 3, fibrinogen, and factor V

PTX3	HVP	PVP	SVP
Bilirubin	0.199 (34)	0.247 (35)	0.086 (34)
Prothrombin time	− 0.361* (34)	− 0.516** (35)	− 0.270 (34)
Antithrombin 3	− 0.184 (30)	− 0.365* (31)	− 0.256 (30)
Fibrinogen	− 0.204 (33)	− 0.297 (34)	− 0.206 (33)
Factor V	− 0.398* (25)	− 0.397* (25)	− 0.311 (25)

Numbers in brackets indicate the number of patients where these data were available. *p < 0.05, **p < 0.01

### PTX3 in patients with hepatocellular carcinoma

Liver cirrhosis is a risk factor for hepatocellular carcinoma (HCC), which is a contraindication for TIPS [6]. In HCV-related HCC patients, PTX3 was high compared to non-cancer patients with mild or severe liver fibrosis [17]. Here, PTX3 was measured in serum of HCC patients where etiology was linked to NASH in 15 patients, hepatitis B infection in 3 patients, alcohol in 5 patients, and remained cryptic in 8 patients. Unfortunately, plasma was not available from this cohort. Measurement of plasma and serum from the same donor yielded higher values in serum [37]. Therefore, PTX3 levels of the HCC and cirrhosis patients could not be compared.

**Fig. 4:**
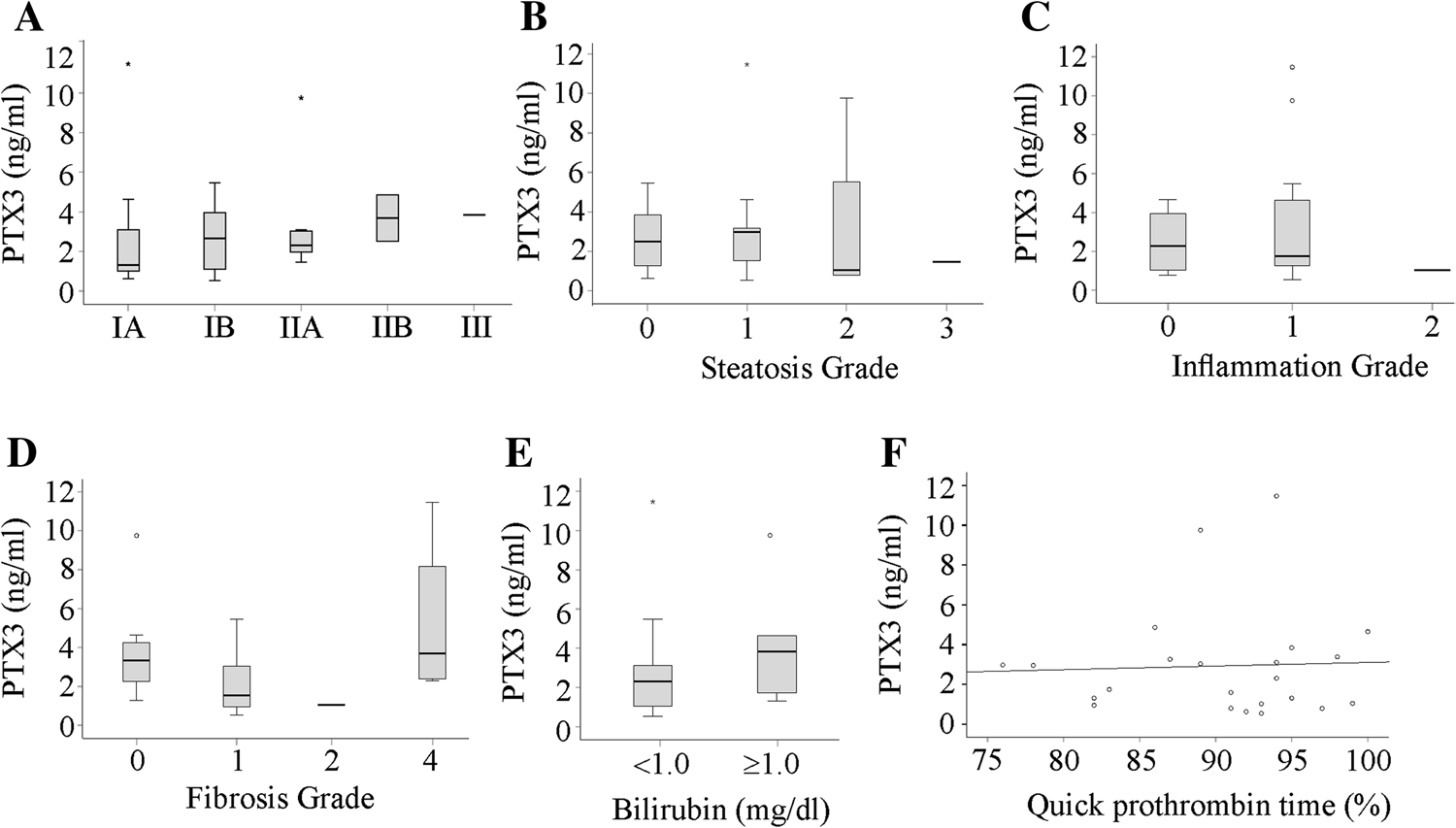
Serum PTX3 in HCC patients. **a** Correlation of PTX3 with TNM classification (TNM stage: IA/IB/IIA/IIB/III, number of patients 13/8/7/2/1). **b** PTX3 in patients stratified for hepatic steatosis (13 patients: no steatosis; 11 patients grade 1; 4 patients grade 2 and 1 patient grade 3; steatosis grade of 2 patients was not known). **c** PTX3 in patients stratified for hepatic inflammation (4 patients: no inflammation; 17 patients grade 1 and 1 patient grade 2. Inflammation grade of 9 patients was not known). **d** PTX3 in patients stratified for hepatic fibrosis (8 patients: no fibrosis; 18 patients grade 1; 1 patient grade 2 and 4 patients grade 4). The number of patients in some subgroups was 1, and statistical test is not appropriate. **e** PTX3 in patients with normal and high (≥ 1) bilirubin. **f** Correlation of PTX3 with Quick prothrombin time

HCC patients were older and had lower bilirubin and improved prothrombin time than cirrhosis patients (Table 1). Serum PTX3 was not related to age, body mass index, or gender (data not shown). Moreover, there was no association of serum PTX3 with tumor node metastasis (TNM) classification in HCC patients (Fig. 4a). In the HCC cohort, 16 patients had histology confirmed liver steatosis, 18 patients had liver inflammation, and 23 patients liver fibrosis. Serum PTX3 was not associated with any of these histological grades (Fig. 4b–d). Moreover, serum PTX3 was not related to bilirubin or prothrombin time (Fig. 4e, f).

## Discussion

Main findings of the present study are that levels of PTX3 (1) were higher in the portal as compared to the hepatic vein (2) and were increased after TIPS insertion. This suggested that the liver eliminates PTX3 from the blood.

PTX3 did not correlate with resistin and galectin-3, which are produced by immune cells [35]. Though circulating PTX3 in patients with liver cirrhosis was supposed as a marker of immune cell activation, high levels are also attributed to diminished hepatic excretion.

Negative correlations of PTX3 with prothrombin time were in accordance with a function of PTX3 in the extrinsic pathway of coagulation [38, 39]. Tissue factor initiates extrinsic blood coagulation and PTX3 enhanced the expression of this protein in activated monocytes and endothelial cells [38, 39]. Thus, shorter prothrombin time in cirrhotic patients with high PTX3 may be because of higher tissue factor expression.

PTX3 further negatively correlated with antithrombin 3, factor V, and fibrinogen. PTX3 indeed binds fibrinogen and enhances plasmin-induced fibrinolysis [10]. Thus, PTX3 promotes fibrin deposition and fibrinolysis, and therefore, has a regulatory function in coagulation pathways [10, 39]. Several pro- and anticoagulant factors are produced in the liver, and consequently, both pathways are impaired in patients with liver cirrhosis [40]. PTX3 was higher in cirrhotic patients than controls, whereas prothrombin conversion as well as thrombin inactivation was impaired in these patients [21, 40]. This suggests that raised PTX3 in liver cirrhosis may in part compensate for deficiencies in pro- as well as anticoagulatory pathways. Correlations of PTX3 with antithrombin 3 and factor V cannot provide information on a functional association of these proteins nor does it prove whether PTX3 directly regulates the hepatic synthesis or the excretion of these proteins.

Negative correlations of PTX3 with fibrinogen and antithrombin 3 were also described in patients with acute Puumala hantavirus infection, a disease mainly characterized by renal dysfunction [41]. In contrast, such associations were not identified in patients with sickle cell painful crisis and women with preeclampsia [42, 43]. Additionally, serum PTX3 did not correlate with prothrombin time in HCC patients. Associations of PTX3 with measures of coagulation were related to pathological condition, suggesting a complex regulatory network. PTX3 was correlated with inflammation, dyslipidemia, and renal function, illustrating that multiple influential factors are involved [7, 13, 14, 44].

PTX3 was not associated with the severity of liver disease defined by the Child–Pugh or MELD score in the cohort analyzed herein. A separate study described modest positive associations of PTX3 with these measures. Mean MELD score in that study was nearly twofold higher than of the patients enrolled in the present investigation. Indeed, patients with a MELD score over 20 had higher systemic PTX3 than patients with a lower score [21]. However, similar to our findings, PTX3 levels were not different in Child–Pugh class A, B, and C patients [21]. Hence, PTX3 is not associated with the severity of liver disease. High levels are more likely related to severe complications such as acute-on-chronic liver failure or infections [21].

This suggestion was supported as circulating PTX3 showed no association with histological liver steatosis, inflammation, or fibrosis in patients with HCC. Of note, serum PTX3 did not increase with the TNM stages in liver cancer. This shows that high plasma PTX3 in HCC, which was even considered as a risk factor for HCC development [17], has no prognostic value.

In cirrhosis patients, PTX3 was neither related to ascites volume nor to variceal size. Moreover, PTX3 levels were not induced in patients presenting with ascites or grades III/IV encephalopathy [21]. This excludes PTX3 as a marker of residual hepatic function and common complications of liver cirrhosis. PTX3 was, nevertheless, higher in patients with variceal bleeding compared to those with refractory ascites. These two cohorts had similar MELD score, prothrombin time, bilirubin, CRP, and resistin (data not shown). Related to the function of PTX3 in coagulation, tissue injury was associated with higher local and systemic PTX3 in a skin-wounding model [10], and similar mechanisms may account for higher PTX3 in patients with variceal bleeding.

Moreover, pathological bacterial translocation is aggravated in patients with variceal bleeding [45]. This boost of proinflammatory gut-derived mediators may further impact on PTX3 levels.

Interestingly, PTX3 was higher in the portal compared to the hepatic vein. PTX3 is mostly released by immune cells, and thus, the spleen and even bowel or omental adipose tissue localized cells may contribute to portal vein PTX3. Median ratio of portal to hepatic vein PTX3 was 117% in Child–Pugh A, 107% in Child–Pugh B, and 100% in Child–Pugh C patients. Though these values did not significantly differ, they tend to decline in patients with more impaired hepatic function. PTX3 may be eliminated by the liver and impaired liver function may thus contribute to higher systemic levels. In line with this assumption, PTX3 levels increased after TIPS. The more prominent rise of PTX3 in HVP compared to PVP shortly after TIPS further supports this hypothesis. A clinically interesting question is whether increased PTX3 shortly after this intervention contributes to higher thrombosis risk [5].

Post-TIPS PTX3 in the portal vein still correlated with prothrombin time, antithrombin 3, and factor V. In HVP, associations with prothrombin time and factor V persisted after TIPS. This principally points to a relatively strong association of PTX3 with coagulation. SVP PTX3 before and after TIPS showed now associations with coagulation factors or prothrombin time. This illustrates that, despite the high correlation of PTX3 levels among each of the blood compartments, additional mechanisms exist that specifically modulate its systemic concentration. Whether coagulation factor levels vary in the distinct blood compartments needs further analysis.

There are limitations within our study. The cohort sizes were low, and effects were rather small. Plasma of HCC patients to analyze PTX3 was not available, and PTX3 levels of the cohorts could not be compared.Moreover, MELD score of HCC patients was not documented.

In summary, the present study provides evidence that the liver eliminates PTX3 from the blood. Impaired hepatic removal of PTX3 in liver cirrhosis may contribute to increased plasma levels. Anyhow, circulating PTX3 is not of prognostic value in liver cirrhosis or HCC.
